# Design Variation, Implantation, and Outcome of Transcatheter Mitral Valve Prosthesis: A Comprehensive Review

**DOI:** 10.3389/fcvm.2021.782278

**Published:** 2022-02-24

**Authors:** Faizus Sazzad, Jimmy Kim Fatt Hon, Kollengode Ramanathan, Jie Hui Nah, Zhi Xian Ong, Lian Kah Ti, Roger Foo, Edgar Tay, Theo Kofidis

**Affiliations:** ^1^Yong Loo Lin School of Medicine, National University of Singapore, Singapore, Singapore; ^2^Department of Cardiac, Thoracic and Vascular Surgery, National University Hospital, Singapore, Singapore; ^3^Cardiovascular Disease Translational Research Programme, Centre for Translational Medicine, National University of Singapore, Singapore, Singapore; ^4^National University Heart Centre, National University Health System, Singapore, Singapore; ^5^Asian Heart & Vascular Centre (AHVC), Mount Elizabeth Medical Centre, Singapore, Singapore

**Keywords:** transcatheter, transcutaneous, systematic review, mitral valve replacement, transcatheter mitral valve implantation (TMVI)

## Abstract

The transcatheter mitral valve prosthesis is ideally suited for patients with inoperable mitral etiology. The transcatheter mitral valve implantation (TMVI) procedure has closely followed the evolution of transcatheter aortic procedures. There are considerable design variations amongst the limited TMVI prostheses currently available, and the implantation profiles of the devices are notably different. This comprehensive review will provide an overview of the current clinically tried TMVI devices with a focused outcome analysis. In addition, we have discussed the various design characteristics of TMVI and its associated failure mode, implantation technology, delivery methods, first-in-man trials, and pivotal trial summary for the synthesis of recent evidence.

**Systematic Review Registration:**

https://www.crd.york.ac.uk/prospero/display_record.php?ID=CRD42021255241, identifier: CRD42021255241.

## Introduction

Mitral regurgitation (MR), also known as mitral insufficiency or incompetence, is a leading cause of death worldwide; in western countries, where rheumatic heart disease has a low prevalence (1), MR remains the most common heart valve pathology. Mitral stenosis (MS) commonly arises as a complication of untreated streptococcus infections, leading to corrective treatment procedures to repair or replace the mitral valve. Treatment options for mitral valve pathologies largely depend on the pathophysiology involved, where significant mitral valve apparatus dysfunction may be apparent due to primary etiology. Structural and coaptation failure of leaflets may be predominantly due to left ventricular remodeling from other etiologies (2, 3).

Transcatheter mitral valve replacement is an evolving treatment for MR that closely follows the success of transcatheter aortic valve replacement (TAVR). Transcatheter mitral valve treatment (TMVT) shows *un avenir prometteur* since its inception (4), despite having several disenchanting limitations (5). TMVT comprises either repair or replacement of the mitral valve; the former has proven its cost-effectiveness and was projected to increase life expectancy in carefully selected cases (6), whereas the latter is yet to break ground. Structural heart valve interventions utilizing transcatheter mitral valve implantation (TMVI) have yet to reach the current therapeutic gold standard compared to surgical mitral valve implantation (SMVI) (7). In contrast, the therapeutic focus of the current transcatheter mitral valve implantation (TMVI) is on patients who are deemed surgically inoperable (8), especially in patients with severe MR and high surgical risk. There is a different therapeutic allocation that has been suggested for primary and secondary MR in the current guidelines (9). Surgical mitral valve repair remained preferred for primary MR when a durable repair is anticipated, whereas transcatheter edge-to-edge repair is preferred over surgery for secondary MR (9). However, transcatheter mitral valve repair (TMVr) and medical therapy together compared to medical therapy alone for severe secondary MR did not show any significant difference in death rate or unplanned hospitalization for heart failure at 1 year (10).

The prosthesis design of the current TMVI devices may hinder the heart's dynamic function, especially during diastole; it may also lead to increased transmitral gradient and reduced effective orifice area (EOA), leading to clinical problems (11). Left ventricular outflow tract obstruction (LVOTO) and annular dimensions outside manufacturer specified ranges are generally recognized as the most frequent reasons for screen failure for TMVI (12). Anchoring of the device onto a nonfibrous mitral annulus exaggerates its migration potential (13), whereas potential LVOTO (14) and interfacing with next-door aortic prosthesis remains a prominent concern (15). On the other hand, the presence of a SAVR/TAVR seems to be less of an issue with some TMVI systems (16).

Additionally, prior TMVr procedure addressing the valve leaflets limits the use of the TMVI device despite the potential necessity. Currently, mini-thoracotomy and transapical TMVI access remain mainstream in approaching the native mitral valve (17). The delivery approach of the device is yet to be explored in elderly patients with poor ventricular ejection. Concomitant surgically viable tricuspid regurgitation (TR) and coexisting atrial fibrillation (AF) cases are not rare and may deserve simultaneous attention. However, severe mitral annular calcification (MAC) and inoperable patients have been successfully treated with balloon-expandable aortic transcatheter valve in the mitral position (18). TMVI with a dedicated prosthesis from Abbott Inc in severe MAC showed early feasibility and MR relief with symptom improvement (19).

Herein, we provide a comprehensive overview of the TMVI devices focusing on the clinical outcomes of first-in-men (FIM) clinical trials. In addition, we discuss the design variation and optimization potential with failure modes of the devices, as required for regulatory approval.

### Evolution of Heart Valve Prosthesis for Mitral Position

Inspired by an old bottle stopper, Albert Starr developed the “Ball-cage” prosthesis design, which was the first mechanical heart valve implanted in the mitral position in 1961 (20). The Starr–Edwards prosthesis faced hemodynamic turbulence due to the absence of central forward flow in the presence of the ball (21); moreover, the more extensive valve profile led to LVOTO and a significant reduction of EOA. The tilting disk, or Björk-Shiley Delrin (BSD) valve, was first introduced in 1969 (22). However, it failed to achieve a physiological central flow pattern. Finally, Kalke and Lillehei developed the rigid bileaflet valve, which was introduced clinically in 1977 by Manny Villafaña's St Jude Medical and implanted by Demetre Nicoloff (23).

The biological valve was a cause of concern due to inert complications recognized from the metallic devices. The biological valve has its advantages in terms of better biocompatibility with questioned durability. The porcine formalin-fixed xenografts were first used by Carpentier in the mitral position in 1965 (24), shortly after they suggested glutaraldehyde for the chemical treatment of porcine valves (25). In 1966, Carpentier designed the stented mitral prosthesis by mounting the whole porcine heart valve into a stent (26). Since then, the central forward flow profile was established; however, stent-related complications led to leaflet stiffness, and glutaraldehyde pretreatment caused dystrophic calcification. Ionescu and associates introduced pericardial heart valves in 1971 using bovine pericardium (27), which was the first complete biological heart valve utilizing bovine pericardium. Ionescu-Shiley valve had the first “tri-leaflet Mercedes-Benzes star” pattern; however, it faced structural valve deterioration (SVD) due to the Delirin flexible stent within the initial five years of implantation (27). Tirone David, in 1988, proposed “stentless valves” without any metal stent and sewing ring for the aortic position. While this technology did not prove superior (28), it paved the way for transcatheter valves and “sutureless” prostheses.

The first catheter-based procedure for mitral stenosis was percutaneous balloon mitral commissurotomy (PBMC); Inoue first used trans-femoral and trans-septal balloons in 1984 (29), which has now become the management of choice for rheumatic MS with pliable valve (30). Bonhoeffer did the first transcatheter heart valve implantation in 2000 in the pulmonary position (31), which was duplicated by Cribier in 2002 for the aortic position (32). In 2003, some devices were utilized to modify Alfieri's surgical edge-to-edge leaflet repair technique to translate it into the catheter-based procedure. Subsequently, the first MitraClip was implanted in Caracas, Venezuela, by Dr. Jose Condado (33).

**Figure 1:**
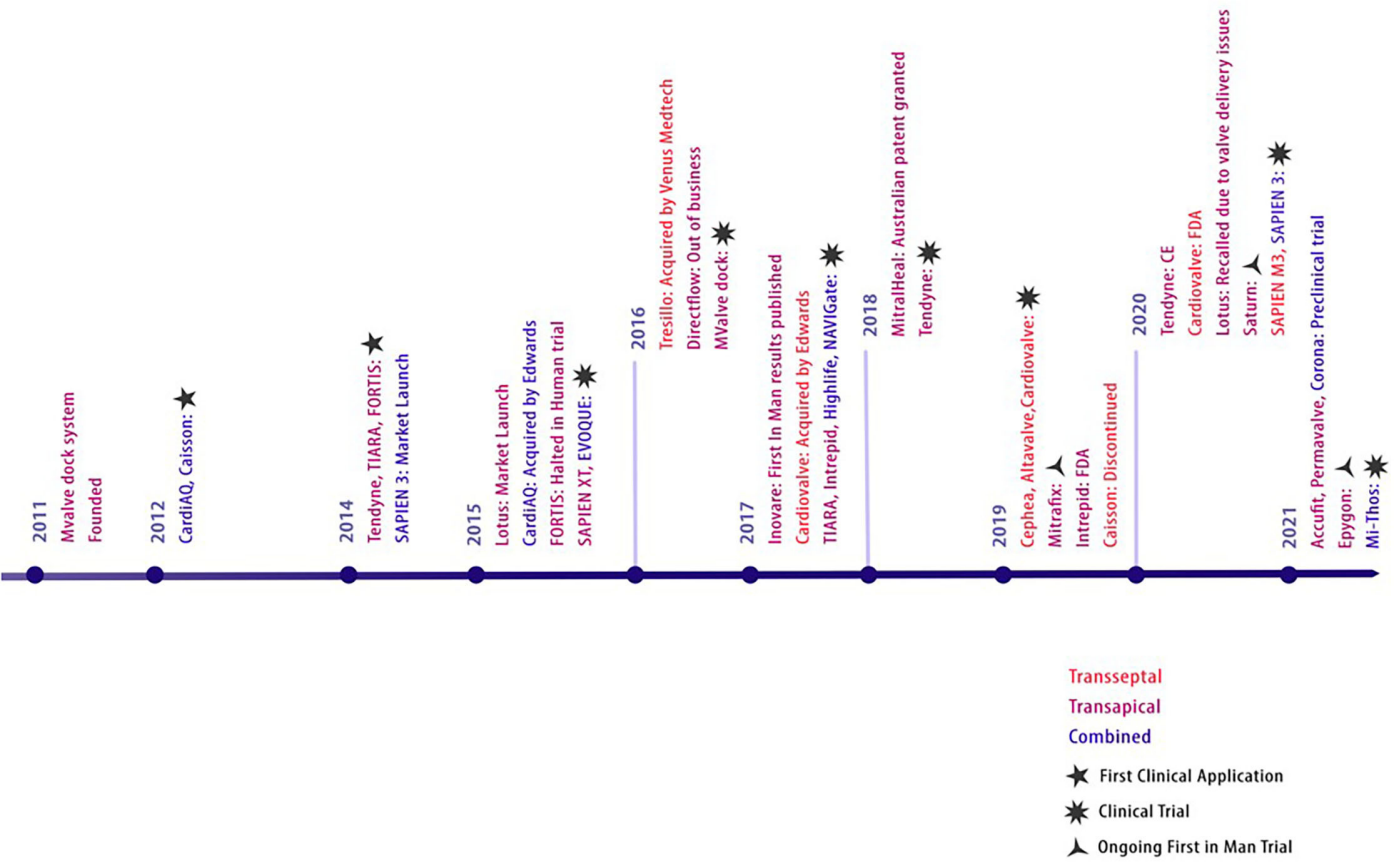
Evolution of TMVI devices. Evolution of transcatheter prosthesis for mitral valve replacement.

The CardiAQ prosthesis was the first dedicated TMVI device that was conceived by Søndergaard in Denmark, 2012 (34). TMVI devices intended for the native mitral valve were rather primitive and faced anchoring and dislodgement issues. The earliest TMVI for native mitral valve stenosis was an inverted transcatheter aortic valve prosthesis (35) *via* transseptal access. Transapical catheter-based procedures were subsequently extended to treat degenerated bioprosthetic valves with valve-in-valve and valve-in-ring procedures by 2014 (36). This required a mini-left thoracotomy for transapical access to implant the Edwards Sapien XT prosthesis. Thereafter, Guerrero pushed the limits to complete a solely percutaneous transfemoral-access-based TMVI in 2014 (37), the first-ever reported in humans to treat severe MAC. The timeline and evolution of TMVI devices are depicted in Figure 1.

## Methods

We conducted a database search for published literature electronically using the preferred reporting items for systematic reviews and meta-analyses (PRISMA) guidelines (38). The literature search extracted records on Medline (*via* PubMed), Embase, Cochrane Library, Scopus, and Web of Science from inception to May 30, 2021.

A repetitive and exhaustive combination of the following “medical subject headings” (MeSH) were used: “mitral valve replacement,” “mitral valve implantation,” “transcatheter,” “transcutaneous,” “systematic review,” “transcatheter mitral valve replacement,” “transcatheter mitral valve implantation”. This study protocol has been registered with PROSPERO (CRD42021255241). Relevant articles were screened and systematically assessed, applying inclusion and exclusion criteria.

### Eligibility Criteria

Studies were only included if published in English, and any experimental cohort studies in humans reporting first and early clinical trials reporting the use of TMVI for mitral valve disease were included. Valves designed and implanted in other cardiac positions were beyond the scope of this study. Mitral valve repair devices for TMVr were also beyond this scope. Furthermore, only studies published after 2010 were included to prevent using nonrelevant data. Articles with TMVI and other concomitant cardiac procedures and nonclinical *in vitro* experiments and animal experiment studies were excluded.

### Study Selection

The extracted citations were screened and assessed by using the reference manager software EndNote X9 independently for inclusion. The articles were first screened by their titles and abstracts, where criteria were purposely broadened to include all relevant studies. Second stage review for studies that have made it through the first stage, or cases where a decision cannot be made, full-text reviews were performed on articles to confirm the relevance. To improve the sensitivity, we have used citation chasing in Google Scholar and Medline (*via* Pubmed). Further data were sought by manual search using the backward snowballing method.

### Data Abstraction and Outcomes of Interest

Three authors independently abstracted details of the study characteristics, TMVI device characteristics, delivery access, periprocedural outcomes, 30 days results, and follow-up, and up to 2 years of data were measured. Data synthesis was done utilizing the ReviewManager 5 software (RevMan 5.4) (39). Depending on the nature of the clinical outcomes extracted from the scientific journals, they were categorized either under dichotomous or continuous data type to generate effect measures. All the results were reported within 95% confidence intervals (CIs).

### Quality of Evidence and Risk of Bias Assessment

All the included studies were prospective observational study with the majority reporting the first-in-men clinical trial. As illustrated in chapter 11 of the Cochrane handbook of reviews (40), GradePro was used to evaluate the quality of evidence in the included studies. Authors assessed the articles for their risk of bias and quality of evidence by using Revmen 5.4. The risk of bias for each study (39) was evaluated according to the guidelines in chapter 8 of the Cochrane handbook of reviews (41).

## Results

**Figure 2:**
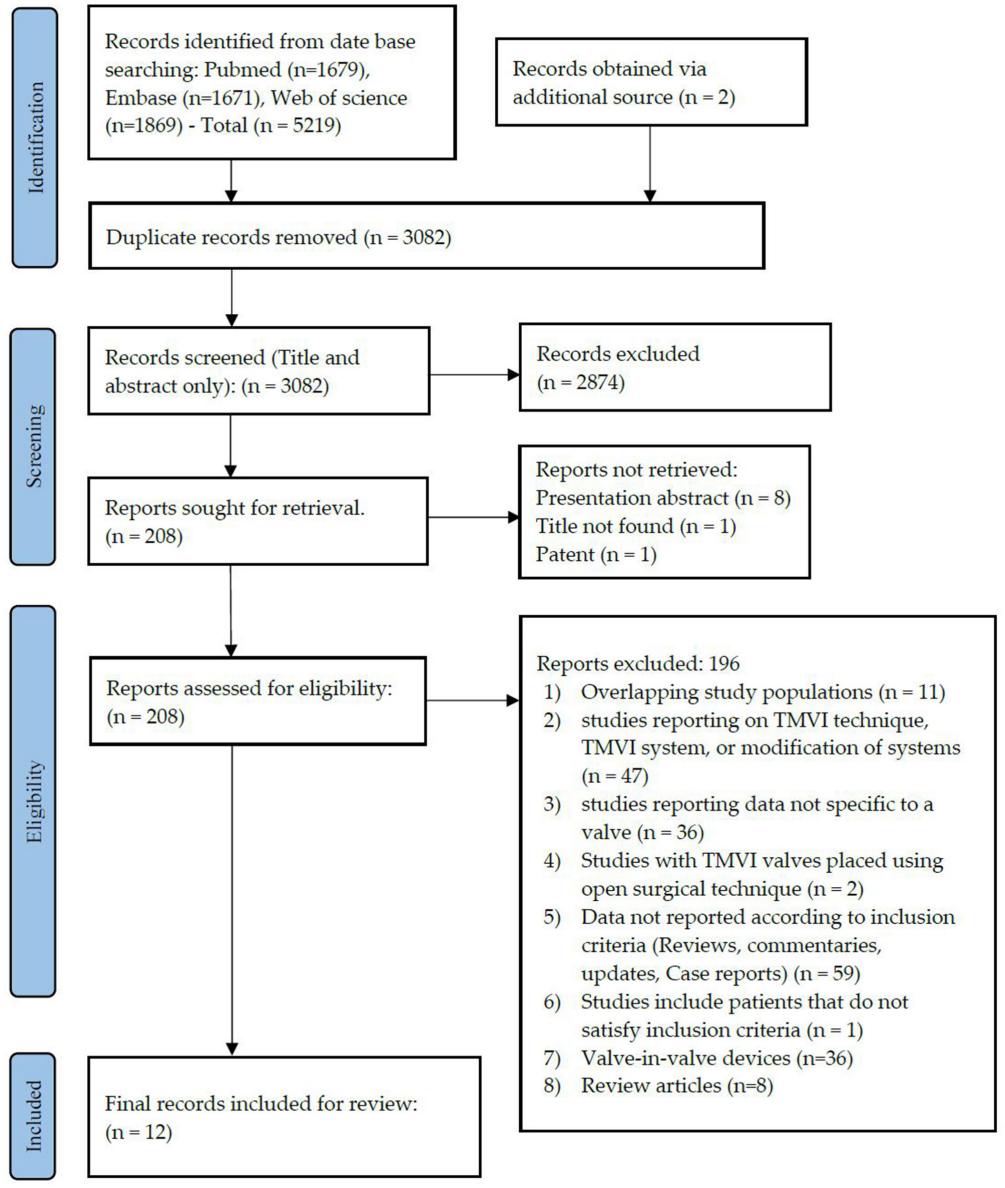
PRISMA flow diagram. PRISMA chart illustrating our process of obtaining the 12 included articles. With 2,874 irrelevant records excluded based on their titles and abstracts, we reviewed the full texts of 208 articles, of which 196 were excluded, and 28 remained for inclusion in our study, of which 12 articles were included for final review.

This systematic search revealed a total of 5,219 articles and systematic reviews. Two other papers were retrieved from alternative sources. After exclusion of the duplicates, 3,082 articles remained for assessment. Irrelevant publications that did not satisfy our inclusion criteria were not considered based on title and abstract scrutiny, leaving 208 articles for full-text review. Following the full-text assessment of these articles, 12 papers (42–53) remained for final review (Figure 2).

**Figure 3:**
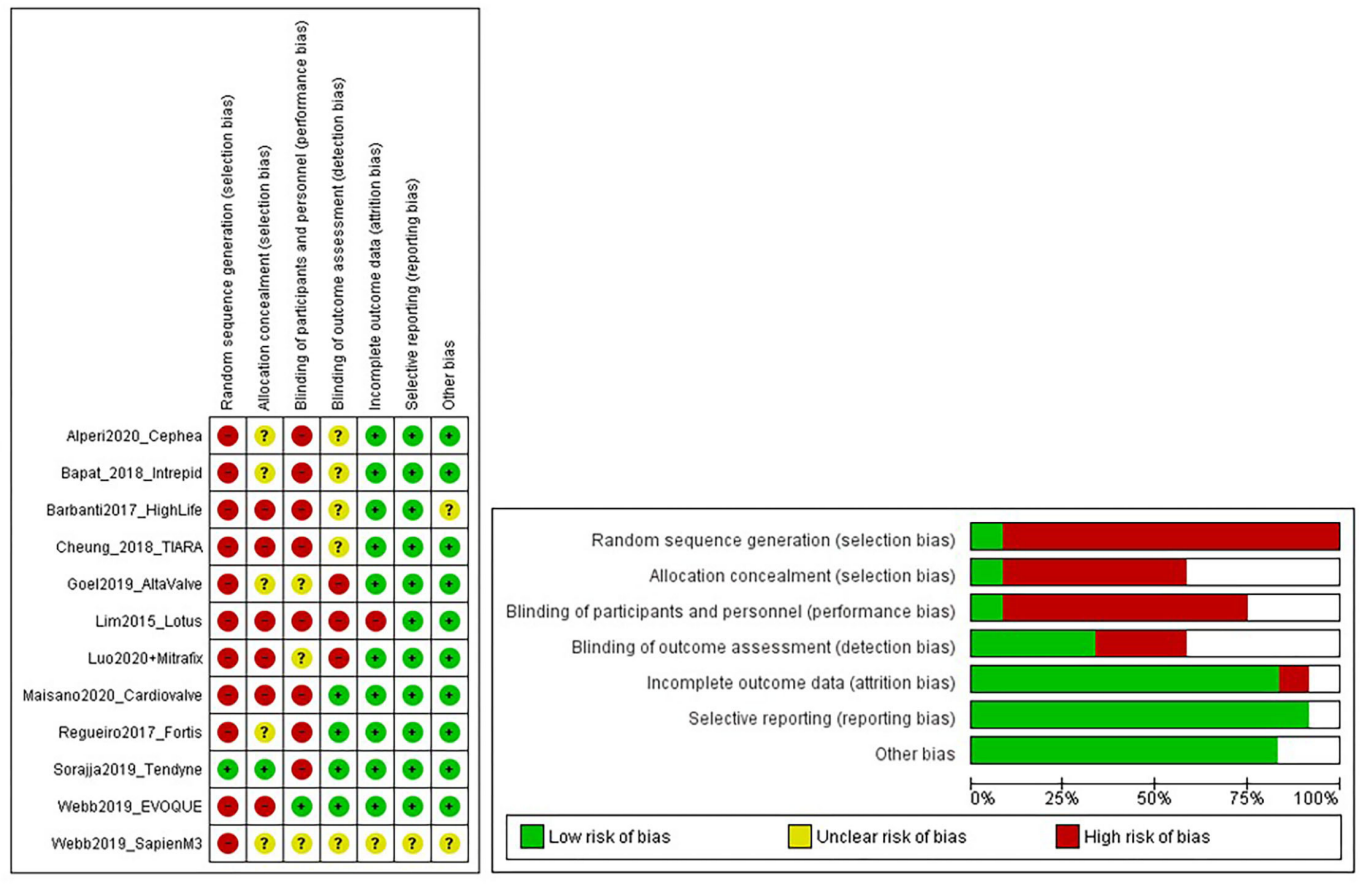
Risk of bias graphs. The figure shows a review of authors' judgments about each risk of bias item presented as percentages across all included studies. Random sequence generation was made high due to selection bias in First-in-man studies.

As seen from Figure 3, the selection bias (54) for each study was critical/important, which we believe can be credited to the type of study itself, the majority being first in the human clinical trial. Despite this, the overall risk of bias for all the studies was classed as low/moderate. Therefore, the evidence provided by these studies was still of acceptable quality.

All studies were First-in-man clinical trials, reporting data on TMVI replacement in single (43, 46, 48–51, 53) or multicenter, global pilot studies (42, 47, 52) with three pivotal trials, namely: Expanded Clinical Study of the Tendyne (NCT02321514), Miscend for Evoque (NCT02718001), and CIP-1403 for Intrepid (NCT02322840). Most of the studies were single-center, with the majority taking place in the United States and Canada. Only the Mitrafix device was from Beijing, China; Cardiovalve in Zurich, Switzerland, and the HighLife was tested in Italy. Two of the 12 included studies (43, 53) in this analysis have been published by the same first author, Webb, in the year (2019) (Table 1). Assessment of the complete texts verified that these studies were performed on two different study populations using two different devices, which have been included separately in our analysis. To aid the identification of these papers, we used the naming Webb et al. (43) and Webb (Late) et al. (53) to differentiate them.

**Table 1:** Summary of the included studies and baseline characteristics of patients.

**References**	**Type of study, device**	**Study period**	**Number of centers: places**	**Number of patients (*n* = 199)**	**Age/Sex**	**NYHA class III/IV**	**Risk Scor EuroSCORE II / STS Score**	**Mitral valve (MR) pathology**	**Associated TR/AF**
Bapat et al. (42)	Clinical trial, Intrepid	May 2015–July 2017	03: Australia, Europe, and USA	50	73 ± 9 (M, 58%)	III: 2 (4.1%), IV: 47 (95.9%)	7.9 ± 6.2 / 6.4 ± 5.5	Primary 8 (16%), Secondary 36 (72%), Mixed 6 (12%)	AF: 29 (58%) ; TR: Moderate 16 (32.7%); Severe 6 (12.2%)
Webb et al. (43)	Clinical trial, SAPIEN M3	Aug 2017–Aug 2018	01: Vancouver, Canada	10	76.1 ± 5.5 (M, 50%)	10 (100%)	5.9 ± 2.2 / 3.8 ± 2.5	Degenerative 4 (40%), Functional 4 (40%), Mixed 2 (20%)	AF: 3 (30%)
Cheung et al. (44)	Case report, Tiara	2018	01: Vancouver, Canada	1	80 (n = 1, M)	1 (100%)	8.40 / 28.4	Functional 1 (100%)	
Barbanti et al. (45)	Case report, HighLife	2017	01: Catania, Italy	2	69, 65 (n = 1, M)	2 (100%)	8.9, 4.5^*^ / -	Functional 2 (100%)	AF: 1 (50%)
Alperi et al. (46)	First-in-man, Cephea	July-Oct 2019	01: Quebec, Canada	3	79 ± 3 (n = 1, M)	3 (100%)	13.8 ± 2.4	Primary 3 (100%)	AF: 2 (66.7%); TR: Moderate 2 (66.6%), Severe 1 (33.3%)
Sorajja et al. (47)	Clinical trial, Tendyne	Nov 2014–Nov 2017	24: Australia, Europe, and USA	100	75.4 ± 8.1 (M, 69%)	99 (99%)	- / 7.8 ± 5.7	Primary 11 (11%), Secondary 89 (89%)	-
Goel et al. (48)	First-in-man, AltaValve	2019	01: Illinois, USA	1	89 (n = 1, M)	-	- / 11.25	-	AF: 1 (100%)
Maisano et al. (44)	First-in-man, Cardiovalve	2020	01: Zurich, Switzerland	1	79 (n = 1, M)	-	-	Functional 1 (100%)	-
Lim et al. (49)	Case report, Lotus	2015	01: London, UK	2	75, 62 (-)	-	-	-	-
Luo et al. (50)	Case report, Mitrafix	2020	01: Beijing, China	2	60, 69 (n = 2, F)	-	- / 10.35, 7.75	-	-
Regueiro et al. (51)	Clinical trial, Fortis	Feb 2014–March 2015	05: Europe and Canada	13	71 ± 8 (M, 76.9%)	-	23.7 ± 12.1^*^ / 7.2 ± 3.6	Secondary 12 (92.3%), Mixed 1 (7.7%)	AF: 8 (61.5%)
Webb et al. (52)	Clinical trial, EVOQUE	Sept 2018 –October 2019	01: Vancouver, Canada	14	84 (79–88.5) (M, 64.3%)	13 (92.9%)	- / 4.6 (3.9–5.6)^#^	Functional 3 (21.4%), Degenerative 4 (28.6%), Mixed 7 (50%)	AF: 13 (92.9)

*NB.:*

* *Logistic EuroSCORE;*

# *Median; AF, Atrial fibrillation; MR, Mitral regurgitation; TR, Tricuspid regurgitation; USA, United States of America, UK, United Kingdom; NYHA, New York Heart Association. All preoperative co-morbidities are summarized in Supplementary Table 1*.

### Basic Demographics

Twelve manuscripts (42–53) that were included reported first-in-man clinical trials and beyond. The majority of the TMVI patients were men and more than 70 years old, with almost all of the patients having a high or a very high surgical risk based on their EuroScore and society of thoracic surgeons (STS) score (Table 1). Preoperative data, namely BMI, 6-min walk test, Kansas City Cardiomyopathy Questionnaire, smoking status, preoperative investigation profile, prior cardiac interventions, and any preoperative extenuating circumstances, are recorded in Supplementary Table 1.

### TMVI Device Design and Access

Design variations, including size, annular shape, anchoring mechanism, access, and recapture features, have been summarized in Table 2. Notably, Tiara (44), Tendyne (47), and Mitrafix (51) devices have a D-shaped annulus, whereas the rest have a circular annulus. Diversity of anchoring mechanism has been observed, namely apical tethers (external anchor), annular winglets, native leaflet engagement, annulus clamping, loading dock system, and radial force. While most devices were implanted *via* apical access (through mini-thoracotomy), some were trans-septal, and the use of both approaches has been observed as well. Several devices were in the pipeline and found to be at different stages of development; this is summarized in Supplementary Table 2.

**Table 2:** Transcatheter mitral replacement devices: Device profile, design, and access.

**Transapical approach (via mini-thoracotomy)**										
**Device**	**Photo**	**Access**	**Valve size**	**Sheath**	**Design**	**Annulus**	**Mounting**	**Anchoring**	**Recapture**	**Status**
Intrepid [Twelve] (Medtronic Inc) (42)^*^	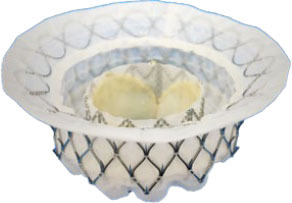	Transapical	27 mm	35 Fr	Self-expanding, tri-leaflet bovine valve	Circular	Mounted on a nitinol frame comprising of an outer and inner stent	Radial force and cleats of the outer frame; the inner frame homes the valve	No	FDA: 2019; NCT03242642
Tiara (Neovasc Inc) (43)^*^	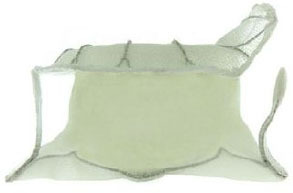	Transapical	35 mm 40 mm	32 Fr 36 Fr	Self-expanding, tri-leaflet bovine bioprosthetic valve	D-shaped	Mounted on a nitinol frame	Radial expansion and ant/post ventricular tabs, atrial flanges.	Partially recapturable (Before ventricular deployment)	Ongoing trial NCT03039855
Tendyne (Abbott Inc) (47)^*^	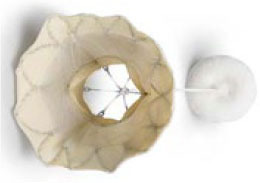	Transapical	34–50 mm (CC) (3 sizes)	34Fr	Self-expanding, tri-leaflet porcine valve.	D-shaped	Mounted on nitinol double-frame stent	The apical pad: It is inserted into position over the tether.	Fully recapturable system after complete deployment	CE: 2020 NCT03433274
Lotus (Boston Scientific) (50)^#^	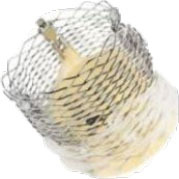	Transapical	23 mm 25 mm 27 mm	20 Fr	Self-expanding, tri-leaflet bovine bioprosthetic valve	Circular	The metallic frame of the valve is braided nitinol.	Implanted into MValve Dock	No	Recalled (2020). Recent FDA approval for the new Lotus Edge
Mitrafix (MitrAssist Lifesciences, Shanghai, China) (51)^≠^	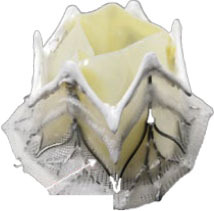	Transapical	35 mm	30 Fr	Self-expanding trileaflet bovine bioprosthesis	D-shaped	Mounted on D-shaped nitinol frame	Atrial flanges with nitinol anchors	No	Ongoing FIM trial ChiCTR: 1900025823
Fortis (Edwards Lifesciences) (52)^*^	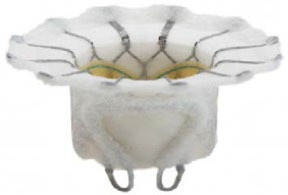	Transapical	29 mm	42 Fr	Self-expanding, tri-leaflet bovine bioprosthetic valve.	Circular	Cylindrical central portion of 29 mm diameter, three leaflets	Mitral valve clipping and paddles. Atrial flanges.	No	Trial halted (mitral regurgitation, thrombus)
Sapien M3 (Edwards Lifesciences) (43)^*^	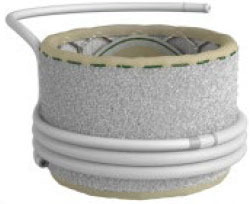	Trans-septal	29 mm	20 Fr	Balloon expandable bovine valve	Circular	Mounted on a cobalt-chromium stent frame.	Nitinol dock encloses native valve and prosthesis together	Retrievable	Ongoing trial NCT04153292
Cephea (Cephea Technologies, Santa Clara, CA) (Abbott Inc) (46)^π^	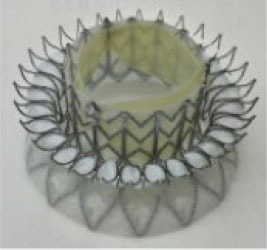	Trans-septal	32 mm 36 mm	36 Fr 38 Fr	Self-expanding, double disk, tri-leaflet bovine bioprosthetic valve	Circular	Mounted on a nitinol frame	Annular anchoring via radial expansion	Partially retrievable	Completed FIM trial NCT03988946
Altavalve 4C Medical, Maple Grove, MN. (48)^Φ^	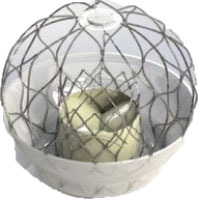	Trans-septal and Transapical	27 mm	30 Fr 34 Fr	Self-expanding, trileaflet bovine bioprosthetic valve	Circular	Mounted on a nitinol single unit stent	Single unit stent expands and anchors in the left atrium	Repositionable and partially retrievable	Ongoing Trial NCT03997305
Cardiovalve (Valtech Cardio Ltd) (Edwards Lifesciences) (49)^*^	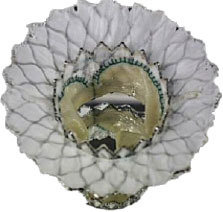	Trans-septal	36–55 mm (CC) (3 sizes)	28 Fr	Self-expanding, tri-leaflet bovine bioprosthetic valve	Circular	Mounted on a nitinol frame	Atrial flanges, annulus anchoring	No	FDA: 2020 Ongoing Trial NCT03813524
**Hybrid approach**										
HighLife HighLife Medical (CA) (45)^*^	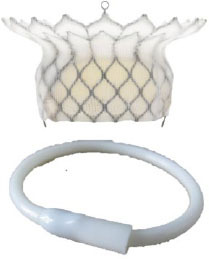	Transapical Trans-septal Transfemoral	31 mm (length of the SAI ring)	39 Fr 18 Fr (SAI)	Self-expanding, tri-leaflet bovine bioprosthetic valve	Circular	Mounted on a nitinol frame. Additional subannular implant	Atrial and ventricular flanges, subannular ring implant	No	Ongoing trial NCT02974881
CardiAQ/ Evoque (Edwards Lifesciences) (53)^*^	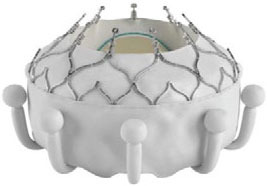	Transapical Trans-septal	44 mm 48 mm	28 Fr	Self-expanding, tri-leaflet bovine bioprosthetic valve with an intra-annular sealing skirt	Circular	Mounted on a nitinol frame	Ventricular anchor, annular attachment, leaflet engagement	No	Ongoing trial NCT02718001

*FDA, Food and Drug Administration; NCT, ClinicalTrials.gov identifier; Fr, French; CE, Conformitè Europëenne; FIM, First in Man; ChiCTR, Chinese Clinical Trial Registry; CC, Inter-commissural distance; SAI, Sub-Annular Implant. Images courtesy*.

* *Reprinted from Front. Cardiovasc. Med. Gheorghe L, Brouwer J, Wang DD et al. Current Devices in Mitral Valve Replacement and Their Potential Complications. 7:531843. © 2020. Frontiers Media SA*.

# *Reprinted from J Am Heart Assoc. Testa L, Popolo R.A, Casenghi M, et al. Transcatheter Mitral Valve Replacement in the Transcatheter Aortic Valve Replacement Era. 19;8(22):e013352. © 2019. © American Heart Association*.

≠ *Reprinted from JACC Cardiovasc Interv. Clinical Application of a Fully Ultrasound-Guided Transapical Transcatheter Mitral Valve Replacement Device. 14;13(17):e161-e162. © 2020. Published by Elsevier*.

π *Reprinted from J Am Coll Cardiol. Regueiro A, Granada JF, Dagenais F et al. Transcatheter Mitral Valve Replacement: Insights From Early Clinical Experience and Future Challenges. 2;69(17):2175-2192. © 2017 by the American College of Cardiology Foundation, Published by Elsevier*.

Φ *Reprinted from JACC: Case Reports. Goel, S. S., Zuck, V., Christy, J., Nallamothu, N et al. Transcatheter Mitral Valve Therapy With Novel Supra-Annular AltaValve. 1(5), 761–764. © 2019. Published by Elsevier on behalf of the American College of Cardiology Foundation*.

### Periprocedural Outcome

The majority of the included studies reported a high procedural/technical success rate, even though each of the device was unique in terms of its design variation; this is summarized in Table 3. The average device implantation time was 40–55 min with a range of 100–235 min of overall procedure time. The contrast volume used in fluoroscopy was 29.1 ± 34.0 ml (47), 41.5 ml (32–94.3) (53), and 72 ± 38 ml (43) as reported. One patient (7.1%) was observed to convert surgery (53) due to moderate to severe PVL. Device-specific procedural complications, namely pericardial effusion [one patient reported by Bapat et al. (42)], bioprosthetic valve dysfunction, and device embolization, were the periprocedural complications reported in the included studies.

**Table 3:** Summary of periprocedural outcome of TMVI devices.

**Device/year**	**Procedure time, min**	**Device time, min**	**Fluoroscopy duration, min**	**Technical success**	**Device retrieval**	**PVL (≥moderate) / ASD closure**	**CPB/IABP support**	**Conversion to surgery**	**Procedural death**
Intrepid, 2018 (42)	100 (80–124)^*^	40 (35–52)^*^		48 (96%)	-	- / -	3 (6%) / 5 (10%)	0	3 (6%)
Sapien M3, 2019 (43)	220 ± 45	-	57 ± 24	9 (90%)	1 (10%)	- / -	- / -	-	-
Tiara, 2018 (44)	-	-	-	1 (100%)	-	0 / -	0 / 0	-	0
HighLife, 2017 (45)	245, 235	-	-	2 (100%)	-	- / -	- / -	-	-
Cephea, 2020 (46)	186 ± 78	-	34 ± 9	3 (100%)	-	0 / 1	0 / 0	0	0
Tendyne, 2019 (47)	136.1 ± 36.3	53.5 ± 15.9	15.3 ± 28.2	96 (96%)	3 (3%)	- / -	0 / 0	-	-
AltaValve, 2019 (48)	-	-	-	1 (100%)	-	- / -	- / -	-	-
Cardiovalve, 2020 (49)	-	-	-	1 (100%)	-	- / -	- / -	-	-
Lotus, 2015 (50)	-	-	-	1 (50%)	1 (50%)	- / -	- / -	-	-
Mitrafix, 2020 (51)	-	-	-	2 (100%)	-	- / -	- / -	0	0
Fortis, 2017 (52)	123 ± 27	54 ± 22	-	10 (76.9%)	-	- / -	- / -	1	0
Evoque, 2019 (53)	179.5 (154.3–206)^*^	44 (40.3–75.3)^*^	-	13 (92.9%)	-	1 (7.1%) / 11 (78.6%)	- / -	1 (7.1)	0

* *Median; PVL, Paravalvular leak; CPB, Cardiopulmonary bypass; IABP, Intra-aortic balloon pump; Procedure time was defined as the initial skin incision to final skin closure; Device time was defined as the duration from apical access to completion of implantation of the valve*.

### Postprocedural Outcome and Follow-Up

Most of the patients were discharged home after successful implantation of the TMVI devices, with a few reintervention during index hospitalization (Table 4). Two patients (2%) from Tendyne (47) reported myocardial infarction. There were few reports of postprocedural cardiac arrest, vascular complications, stroke/transient ischemic attack (TIA), and acute kidney injury (AKI) (1–8%) (46, 47, 53). A rise in the mean mitral gradient was observed from the point of discharge to 30 days follow-up (2.3–6 mmHg on average). Few studies reported 1-year follow-up data, with only one study completing 2 years of follow-up (52) and none reporting on the structural deterioration of the prosthesis (Table 4). All-cause mortality at 1 year was reported as high as 26% for Tendyne (47) and 22% for Intrepid (42). Two years all-cause mortality was only reported by Fortis as 15.4% (52).

**Table 4:** TMVI-postoperative up to 30 days outcome and follow-up variables.

**Device**	**ASD closure**	**Mean mitral gradient, mm Hg**	**New-onset AF**	**Length of hospital stay, days**	**LVEF at discharge**	**LVOTO**	**Re-intervention related to MV**	**MR grade <1 (30 days)**	**Mean mitral gradient, mm Hg (30 days)**	**All-cause mortality (30 days)**	**Kansas city cardiomyopathy questionnaire 12 (1 year)**	**All-cause mortality (1 year)**	**All-cause mortality (2 years)**
Intrepid (42)	-	-	7 (14%)	-	36.2 ± 10.2	0	-	8 (19%)	4.1 ± 1.3	7 (14%)		11 (22%)	-
Sapien M3 (43)	-	2.3 ± 1.4	-	1.5^*^	33 (30, 45)	0	-	6 (66.7%)	6 (5–6)^*^	0	-	-	-
Tiara (44)	-	2	-	-	-	-	-	-	-	-	-	-	-
HighLife (45)	-	4–6	-	-	-	-	-	2 (100%)	-	-	-	-	-
Cephea (46)	1 (33.3%)	3 (2–4)	-	9 ± 5	53.7 ± 1.2	-	-	1 (33.3%)	2.3 ± 0.2	0	72.4 ± 15.7	0	-
Tendyne (47)	-	-	2 (2%)	11.1 ± 8.7	-	-	1 (1)	-	-	6 (6%)	>5 = 81.3%, >10 = 73.4%	26 (26%)	-
AltaValve (48)	-	1	-	-	-	0	-	-	-	1	-	-	-
Cardiovalve (49)	1 (100%)	5	-	-	-	-	-	-	-	-	-	-	-
Lotus (50)	-	4,7	-	6	-	-	-	-	-	-	-	-	-
Mitrafix (51)	-	-	-	-	53.5 ± 6.5	-	-	-	-	0	-	-	-
Fortis (52)	-	2.6 ± 1.1	1 (7.7%)	-	-	0	1	-	-	5 (38.5%)	-	-	2 (15.4%)
Evoque (53)	-	3 (2–4.3)^*^	-	-	43.5 (38–51.5)^*^	1 (7.1%)	-	12 (100%)	5.8 (5–6.8)^*^	1 (7.1%)	-	-	-

* *Median*.

*ASD, Atrial septal defect (caused by trans-septal device); AF, Atrial fibrillation; LVEF, left ventricle ejection fraction; LVOTO, Left ventricle outflow tract obstruction; MR, Mitral regurgitation; MV, Mitral valve*.

### Secondary Outcome

The average age of the included patients was >75 years with high STS [>7.4%, IQR: 3.8%−11.25%] and EuroScore-II [>14, IQR: 5.9–28.4]. Most of the patients were NYHA III/IV with multiple comorbidities. Around 38% of the patients had prior coronary artery bypass graft surgery (CABG), and 10% had other cardiac procedures, as Bapat et al. (42) reported. Most of the included TMVI devices were indicated for severe degenerative MR patients with a high surgical risk. There were associated moderate to severe unaddressed TR (42, 46) and atrial fibrillation (42, 43, 45, 46, 48, 52, 53) in some studies, for which no surgical intervention was reported. The durability of the devices could not be commented upon due to the lack of data beyond 2 years of follow-up.

## Discussion

Designing a TMVI device is a challenging task, and the design variation of the currently available devices shows the magnitude of the anticipated difficulties. Various new stent design approaches were developed to address LVOTO, valve anchoring, sealing, and dynamic deformation of the mitral annulus throughout the cardiac cycle. The key findings of this systematic review are been summarized in Table 5.

**Table 5:** Key findings.

**Key points**	**Summary**
SMVI	Surgical mitral valve implantation is the standard of care for patients requiring a mitral valve replacement. Transcatheter mitral valve implantation (TMVI) is an evolving technology that provides a notable alternative for patients with uncertain outcomes anticipated.
Pathology	MV pathology is varied. So unlike surgery, there is unlikely to be a 'one valve fits all' percutaneous valve replacement solution.
Anatomy	The native mitral annulus has no calcium, no anchorage (not fibrous), the aortic valve in the vicinity (may have another prosthesis), conduction tissue compromised if overexpanded. These make valve migration, paravalvular leak, LVOTO, need for PPM a real problem.
Access	MV has a larger annulus, and hence larger valves are needed. This problem currently restricts valve delivery to transapical as the predominant delivery route. Doing transapical punctures in frail patients with poor LVEF is not ideal, limiting the progress of such technology.
Age and valve durability	MV patients are, on average, 10 years younger than aortic stenosis patients. Hence, the durability issue will be an essential question to answer before any percutaneous valve replacement will become established if and when they become available.
AF and TV	MV disease usually coexists with TV regurgitation and AF. Most percutaneous mitral valve replacement trials have excluded patients with TR.
Trend	MV technologies though facing headwinds, corroborates with limitations as above; surgical mitral valve replacement remains the gold standard. However, at least 12 devices have been evolved, showed early success in FIM clinical trials.

### LVOTO and Device Size

Prior to a TMVI procedure, each patient undergoes contrast-enhanced cardiac computed tomography to assess the mitral annulus and the left ventricular outflow tract (LVOT). Multiple factors increase the risk of LVOTO, including an obtuse aortomitral angle, degree of septal hypertrophy, and left ventricular size (55). Additionally, the proximity of the aortic leaflet to the LVOT presents a high risk of LVOTO. One of the design strategies includes reducing stent protrusion into the ventricle, which may help to reduce the displacement of the anterior mitral leaflet toward the LVOT (56). Intrepid, for example, has a small device profile of 17 to 18 mm to minimize the risk of LVOTO (42). Similarly, Cephea was designed with a small surgical valve-like profile (46). Altavalve overcame LVOTO *via* its design as a fully supraannular stent (48). Highlife adopts another approach that mainly reduces the risk of LVOTO by accurate positioning of the valve within the subannular implant (SAI) (45).

Suitable valve size was chosen *via* various measurements such as, but not limited to, intercommissural diameter, anteriorposterior distance, and mitral annular perimeter. Adequate sizing draws a balance between oversizing for anchoring and fixation, described in the implantation of Intrepid and Altavalve while minimizing the risk of LVOTO (42, 48). An excessively oversized valve may also risk atrioventricular groove injury.

In the included studies, TMVI devices had sizes varying from 23 and 55 mm, with Tendyne and Cardiovalve each showcasing the most extensive range with three sizes-covering an interpuncture range of 34–50 mm and 36–53 mm, respectively (47, 49). Tiara and Evoque currently provide two size variations, and the rest of the valves have a single size that covers a range of intercommissural distances (44, 53). In comparison, some TMVI devices currently in development like Accufit and NAVI System present many potentially available sizes.

### Design Variation

Accommodation to the dynamic deformation of the mitral annulus during the cardiac cycle is fundamental to procedural success (57). Most TMVI devices have circular valve components housed in an expanding stent frame. Intrepid (42) and Cephea (46) have described stent designs that isolate the valve prosthesis from the outer fixation ring to prevent distortion of the inner valve. Intrepid adopts a double stent design that allows the inner leaflets to maintain circular geometry, whereas the outer ring conforms to the dynamic anatomy of the mitral annulus (42).

At the same time, Cephea has a central column that supports the leaflets and is isolated from external deformation (58). Another design approach has a stent design that accommodates the saddle-shaped mitral annulus. For example, Intrepid (42) and Cephea (46) have circular valve orifices housed in a conformable outer stent, whereas Tiara (44), Tendyne (47), Mitrafix (51) were designed with a D-shaped valve annulus. Natural D-shaped pattern at the annulus design ensures biomimicry and is considered a critical success factor, as seen in Tendyne (59).

### TMVI Anchoring Mechanism

Improper anchoring and positioning of the TMVI device may result in valve migration. The risk of migration into the left atrium is the highest due to the increased cyclic left ventricular systolic pressures. The native mitral annulus lacks a rigid fibrous ring. Where fibrosis or calcification is absent, the anchoring of a new valve is intricate. As such, TMVI devices developed distinct anchoring mechanisms to ensure proper positioning and sealing.

Majority of the devices anchor *via* radial expansion at the annulus level, supported by either atrial flanges, ventricular tabs that grasp the native leaflets, or both. Tiara, for example, presents a tri-fold anchoring mechanism with atrial flanges, two anterior clips that anchor the valve to the aorto-mitral curtain, and one posterior tab that clips the posterior mitral leaflet onto the rear shelf of the mitral annulus, in addition to fixation *via* radial expansion (60). In contrast, Cephea atrial and ventricular disk design anchors the device *via* axial compression forces and avoids subvalvular anchors or tethers (58). Other devices like Tendyne anchors to the LV apex *via* a tether (47), whereas Altavalve anchors fully in the left atrium using a spherical single unit stent (48). Devices, such as Sapien M3 (43) and HighLife (45) anchor to their dock and SAI, respectively. Proper anchoring aimed to prevent valve migration and was reported to be few in a systematic review of >300 cases with documented early experience (61), subject to be evaluated in large-scale clinical evaluation.

### Paravalvular Leak

Multiple design approaches were used to minimize paravalvular leaks in the current devices and may be used concurrently. A standard method within the existing devices (Sapien M3, Tiara, Tendyne, Altavalve, Evoque) includes incorporating an atrial fabric skirt sewn into or outside the expandable nitinol frame (43, 44, 47, 48, 53). Altavalve, for instance, has a polyethylene terephthalate skirt fabric added to the lowest third of the spherical atrial structure that will enhance endothelization and tissue ingrowth, and in doing so, avoid paravalvular leakage (62). A unique approach presented by HighLife includes using the native mitral leaflets as a seal as it is trapped within the interaction between the prosthesis and its SAI (45).

### Valve in Valve or Valve in Ring

Following the promising developments in TAVR devices, some of the earliest and common TMVI procedures were done using TAVR devices (e.g., Sapien M3, Sapien XT) *via* valve-in-valve (VIV) approach in degenerated bioprostheses or severe MAC patients (63–66). This is especially since redo surgery for the degenerated bioprosthetic mitral valve in elderly patients with impaired left ventricular dysfunction and previous cardiac surgery is not ideal (67). The mortality and morbidity from redo surgeries have increased the demand for minimally invasive treatment options like TMVI.

However, the mitral valve is larger than the aortic valve, provides less support, and is much more asymmetric in shape, which has made the use of TAVR devices in the mitral position to valve in ring (VIR) procedure quite challenging. Fortunately, unlike the saddle-shaped native mitral annulus, the old prosthetic valve creates a circular morphology in which devices initially designed for aortic valve replacement can be implanted. Devices such as Sapien M3 were readily available commercially on account of established success in TAVR and are well studied in the literature. Direct flow has currently been discontinued; Inovare and Sapien M3 continue their clinical trials specifically for implantation in the mitral area (68). Mi-Thos and Meril's MyVal are new valves developed for VIV implantation and address prosthesis or conduit dysfunction (69, 70).

### Access and Mode of Delivery

**Figure 4:**
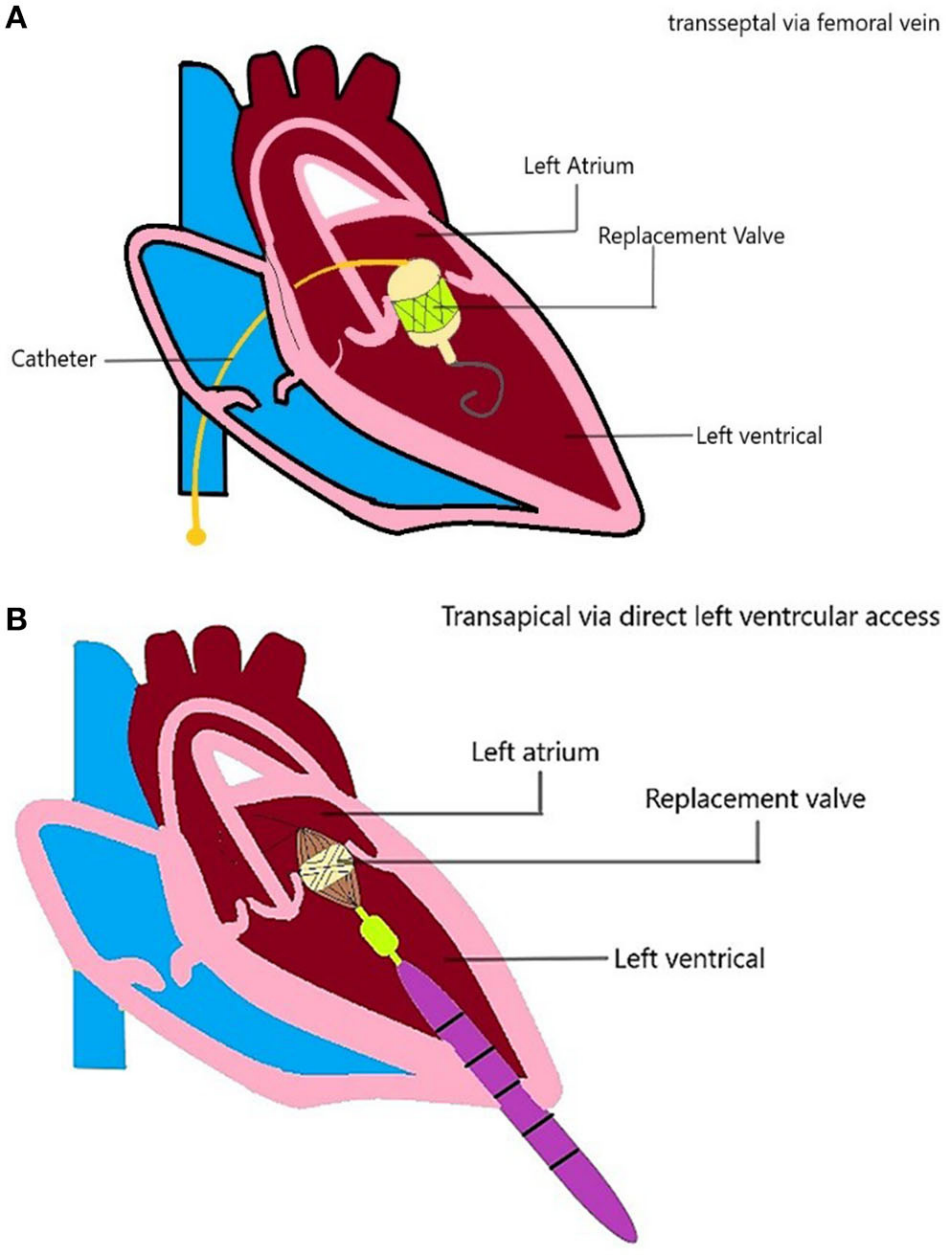
TMVI device access. Schematic diagram showing the TMVI access **(A)** transseptal/transfemoral access, and **(B)** transapical access of implantation.

There are several access strategies for TMVI, including transapical, transseptal, or hybrid (Figure 4). The most common mode of access is the transapical route used for Tiara, Tendyne, Lotus, Mitrafix, Fortis, and Intrepid (discontinued) (42, 44, 47, 50–52). The transapical route gives direct access and ease of maneuvering for coaxial alignment of a larger prosthetic valve to an angled mitral annulus compared to a transseptal approach, and it is favored in early-generation devices (Accufit, Saturn, Epygon, Permavalve, etc.). However, the experience in TAVR has shown that transapical punctures are associated with worse outcomes than transseptal or transfemoral access.

For example, higher degrees of myocardial damage (7), especially in frail or elderly patients (71), and other adverse effects were related to thoracotomy (72). Advances in TMVI technology such as smaller and more flexible delivery catheters have opened the doors for developing a new generation of devices delivered *via* the transseptal route, e.g., Sapien M3, Cephea Altavalve, Cardiovalve (43, 46, 48, 49).

Earlier-generation transapical devices have also been attempted through a transseptal approach, whereas the second-generation CardiAQ/Evoque valve may be implanted *via* either access route (53). Intrepid began its early feasibility trial (2019) for the TMVI system with a transseptal approach after a successful pilot trial (42). The HighLife valve is unique in its approach as it requires two separate access sites (45). The SAI is delivered retrogradely from a transfemoral access site, whereas the valve prosthesis proper is delivered *via* a transapical route.

### Retrievability

Retrieval of a device during or after deployment allows for repositioning and more accurate device placement. Of the TMVI devices, only Tendyne presents a fully retrievable system that can be recaptured after the complete deployment of the device (47). Tiara, Sapien M3 (appears to be discontinued), and Altavalve are partially retrievable and may be recaptured at different stages before fully deploying the device (43, 44, 48). The remaining systems have not been described to be retrievable. However, Intrepid's dual frame structure allows the device to conform to the annulus, whereas the inner frame remains circular and symmetrical has been described to eliminate the need for rotational orientation and thus retrieval (42).

### Crimping Damage

Transcatheter mitral valve implantation systems have an extensive profile and may expose early leaflet damage from crimping (73). Evidence suggests that bioprosthetic valves are subjected to significant surface and deep tissue damage, and reductions in leaflet strength, which may not be reversible (74). With the trend growing toward a transseptal mode of access for TMVI devices, so does the need to crimp TMVI devices into smaller profile delivery catheters, the smallest of which is 20 Fr. Studies based on TAVR devices show that crimping damage to the leaflets increases with smaller delivery profiles and longer crimping duration; however, these concerns and their effects on durability are yet to be explored.

### Durability and Thrombogenicity

Durability is of concern, especially for transcatheter devices to be implanted in younger patients. Studies in aortic valves that examined the durability between transcatheter and surgical prosthetic valves suggest that the durability of both approaches is comparable (75). All of the TMVI devices in the review used bovine and porcine pericardial tissue leaflets. Tissue leaflets underwent accelerated wear tests and have a proven record of passing the ISO 5840-3 with a minimum requirement of 200M cycles. This is the material of choice for prosthetic heart valve manufacturers (76).

While we know that all valves, excluding Epygon, which has a mono-leaflet design, are trileaflet in design, little information has been disclosed about specificities of leaflet design concerning improving durability. The long-term results are yet to be published from a clinical perspective except for a few reporting 2-year outcomes at the best (49, 52). Structural device features such as high metal burden, leaflet material, size, and flow patterns could potentially impact the thrombotic risk. The mode of delivery (transseptal vs. transapical) may also be associated with periprocedural thrombosis. However, data on the risk of long-term thrombosis is scarce, and no dedicated studies have evaluated the thrombogenic profile of the devices concerning these aspects.

## Conclusion

Despite multiple devices showing promising early results, significant advancements have not been achieved for TMVI therapies. Some of the technical challenges gained consensus. Most opinion-leaders favor a transseptal route for delivery, if eventually technically achievable. However, there remain potential challenges. Hence, the “dream” TMVI is a distance from the “Standard of care”. SMVI is still the preferred approach for most patients with mitral valve disease. Importantly, preserving native valve anatomy, selective candidacy, preoperative multimodality imaging, and guideline-directed treatment strategy are still warranted for the transcatheter management of mitral valve pathologies.

## Data Availability Statement

The original contributions presented in the study are included in the article/Supplementary Material, further inquiries can be directed to the corresponding author.

## Author Contributions

FS and JH contributed to conceptualization. FS, ZO, and JN contributed to methodology and data curation. FS and JN contributed to software, validation, writing original draft preparation, visualization, and formal analysis. FS, JH, and KR contributed to resources. FS, KR, ET, and JH contributed to writing and reviewing. JH, LT, ET, RF, and TK contributed to writing and editing. TK managed supervision. FS contributed to project management and administration. FS and TK contributed to funding acquisition. All authors have read and agreed to the published version of the manuscript.

## Funding

The author(s) disclosed receipt of the following financial support for the research and publication of this article. This work was supported by the National Research Foundation (NRF), Singapore, Central Gap Fund (NRF2020NRF-CG001-018).

## Conflict of Interest

The authors declare that the research was conducted in the absence of any commercial or financial relationships that could be construed as a potential conflict of interest.

## Publisher's Note

All claims expressed in this article are solely those of the authors and do not necessarily represent those of their affiliated organizations, or those of the publisher, the editors and the reviewers. Any product that may be evaluated in this article, or claim that may be made by its manufacturer, is not guaranteed or endorsed by the publisher.

## Abbreviations

AF: Atrial fibrillation
ASD: Atrial septal defect
CC: Inter-commissural distance
CE: Conformitè Europëenne
ChiCTR: Chinese Clinical Trial Registry
CPB: Cardiopulmonary bypass
EOA: Effective Orifice Area
FDA: Food and Drug Administration
FIM: First in Man
IABP: Intra-aortic balloon pump
LVEF: left ventricle ejection fraction
LVOTO: Left ventricular outflow tract obstruction
MAC: Mitral annular calcification
MR: Mitral regurgitation
MS: Mitral stenosis
MV: Mitral valve
NCT: ClinicalTrials.gov identifier
NYHA: New York Heart Association.
PMBC: Percutaneous Balloon Mitral Commissurotomy
PPM: permanent pacemaker implantation
PRISMA: Preferred Reporting Items for Systematic Reviews and Meta-Analyses
PVL: Paravalvular leak
SAI: Sub-Annular Implant
SMVI: surgical mitral valve implantation
SVD: Structural valve deterioration
TMVI: Transcatheter mitral valve implantation
TMVr: Transcatheter mitral valve repair
TMVT: Transcatheter mitral valve treatment
TR: Tricuspid regurgitation
TV: Tricuspid valve
VIR: Valve in ring procedure
VIV: Valve in valve procedure
STS: Society of Thoracic Surgeons
AKI: Acute kidney injury
TIA: Transient Ischemic Attack
CABG: Coronary artery bypass graft surgery.
