# Supplementary material for: Design Variation, Implantation, and Outcome of Transcatheter Mitral Valve Prosthesis: A Comprehensive Review
Source: Front Cardiovasc Med. 2022 Feb 24;8:782278. doi: 10.3389/fcvm.2021.782278 (PMC8907442; doi:10.3389/fcvm.2021.782278)
Supplement: Supplementary file 2 [file Data_Sheet_1.PDF]

Supplementary Table-2. Transcatheter Mitral Replacement devices in development

A. Transapical/Transatrial

Device	Photo	Access	Valve size	Sheath	Design	Annulus	Mounting	Anchoring	Recapture	Status
Accufit (Sinomed Innovation)	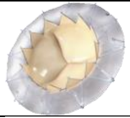	Transapical	30mm<br>34mm<br>38mm<br>42mm	38 Fr	Self-expanding, tri-leaflet bovine bioprosthetic valve.	Circular	The self-expanding, self-centring AccuFit device body has an atrial flange, a ventricular flange	Supra-annular fixation and mitral valve clipping	-	Preclinical study report published
MValve Dock (Mvalve Tech) (Boston Scientific)	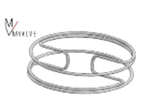	Transapical	-	32 Fr	Boston Scientific Lotus heart valve implanted into MValve docking system	Circular	-	External anchor, mitral annulus capture	-	MValve doc is incorporated with Boston Lotus valve
Saturn (InnovHeart Inc)	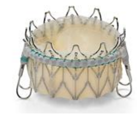	Transapical	-	-	Self-expanding, tri-leaflet, valve	Circular	External annular creation by flexible pair of annular guide wires. The circular central core is made of nitinol frame	Surgical-like anchoring to mitral annulus	No	Ongoing FIM trial
Epygon (Affluent Medical SA)	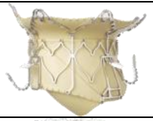	Transapical	-	-	Self-expanding, mono-leaflet pericardial valve	D-shaped	Mounted on an asymmetrical nitinol frame	-	-	Ongoing FIM trial
Permavalve (Micro Interventional Devices, Inc)	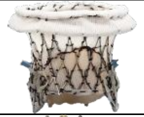	Transapical	-	28 Fr	PolyCor anchors secure the valve to the native annulus.	Circular	-	PolyCor anchoring to the native annulus (subannular hooks)	-	Preclinical
SAPIEN XT (Edwards Lifesciences)	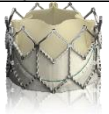	Transapical	20mm<br>23mm<br>26mm<br>29mm	16 Fr	Balloon expandable, tri-leaflet bovine bioprosthetic valve	Circular	Mounted on a nitinol frame	Radial expansion and anchoring to valve	Yes	(VIV device)
Inovare (Braile Biomédica)	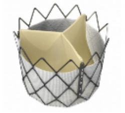	Transapical	20mm<br>22mm<br>24mm<br>26mm<br>28mm<br>30mm	24 Fr	Balloon expandable prosthesis, tri-leaflet bovine bioprosthetic valve	Circular	20mm high Lozenge chromium cobalt frame with three radiopaque markers	Radial expansion	No	Ongoing trial (VIV device)
Direct Flow (Direct Flow Medical)	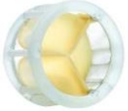	Transapical	25mm<br>27mm	24 Fr	Inflatable bovine bioprosthesis	Circular	Nil mounting	Nil stent	Yes	Out of business (VIV device)
MitralHeal	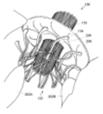	Transapical	NA	NA	Prosthetic valve with at least one arm shaped to deflect chordae tendineae when deployed	Circular	Outer and inner self expanding nitinol frames	Radial expansion and hook-like arms around chordae tendineae	No	Filed for patent (2013) PCT/IL2013/050432 Australia patent granted (2018)

B. Trans-septal approach

Device	Photo	Access	Valve size	Sheath	Design	Annulus	Mounting	Anchoring	Recapture	Approval
Caisson (LivaNova PLC)	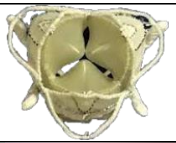	Trans-septal	36mm 42mm	31 Fr	Self-expanding, tri-leaflet porcine bioprosthetic valve	D shaped	Mounted on a nitinol frame	4 subannular “feet” and 3 atrial holding features	Fully recapturable	Discontinued NCT02768402
Transcat. Tech. TRESILLO	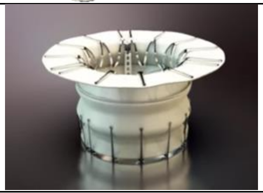	Transseptal	NA	NA	Self expanding trileaflet bovine prosthetic valve	Circular	Nitinol stent	Anchors hook on the native valve leaflets	No	Company acquired by Venus Medtech (2016)

C. Hybrid approach

Device	Photo	Access	Valve size	Sheath	Design	Annulus	Mounting	Anchoring	Recapture	Approval
Sapien 3 (Edwards Lifesciences)	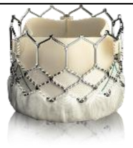	Transapical Trans-septal	20mm 23mm 26mm 29mm	14 Fr 16 Fr 18 Fr 21 Fr	Balloon expandable trileaflet bovine bioprosthetic valve with polyethylene terephthalate (PET) fabric skirt	Circular	Mounted on cobalt-chromium frame	Radial expansion, valve anchoring	Yes	FDA: 2020 Clinical trial NCT03230747. VIV device
NAVI System (NaviGate Cardiac Structures Inc)	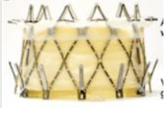	Transatrial Transapical Trans-septal	36mm 40mm 44mm 48mm 52mm	30 Fr	Self-expanding trileaflet equine bioprosthetic	Circular	Mounted on tapered nitinol stent	Atrial winglets and engagement of mitral leaflets and subvalvular apparatus via ventricular graspers	Partial	Ongoing trial FIM for tricuspid position reported
Mi-thos™ valve (NewMed Medical Co., Ltd., Shanghai, China	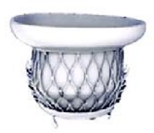	Transapical Trans-septal	-	-	-	Circular	-	-	No	Ongoing trial NCT04195984 VIV device
Corona (Valcare)	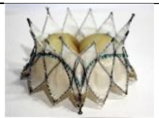	Transapical or Trans-septal	-	21Fr	Self-expanding	D-Shaped	AMEND Mitral repair ring	Ring snapping and Radial force	Not known	Preclinical

FDA= Food and Drug Administration, VIV= Valve-in-valve, NCT= ClinicalTrials*.gov identifier, PCT/IL=Patent Cooperation Treaty/Israel International patent application number, Fr=French, CE= Conformitè Europëenne, FIH= First in human*
